# The Dens: A Review of its Diverse Nomenclature and a Recommended Simplified Terminology

**DOI:** 10.7759/cureus.981

**Published:** 2017-01-17

**Authors:** Jaspreet Johal, Christian Fisahn, Brittni Burgess, Marios Loukas, Jens Chapman, Rod J Oskouian, R. Shane Tubbs

**Affiliations:** 1 Department of Anatomical Sciences, St. George's University School of Medicine, Grenada, West Indies; 2 Orthopedic Surgery, Swedish Neuroscience Institute; 3 Department of Trauma Surgery, BG University Hospital Bergmannsheil, Bochum, Germany; 4 Seattle Science Foundation; 5 Orthopedics Spine Surgery, Swedish Neuroscience Institute; 6 Neurosurgery, Complex Spine, Swedish Neuroscience Institute; 7 Neurosurgery, Seattle Science Foundation

**Keywords:** craniocervical junction, anatomy, terminology, c2 vertebra, odontoid process

## Abstract

Pathology of the dens, such as fractures, demands precise terminology so that communication between physicians are succinct, diagnoses are accurate, and treatment strategies exact. This review aims to summarize the various terms used to describe the parts of the dens and recommend the ideal terminology. Using standard search engines, English language publications were searched for the many terms used to describe parts of the dens. A multitude of terms was identified with many demonstrating overlaps. Terms identified included apex, tip, apicodental, subdental, dentocentral and odontocentral junctions, peg, waist, base, neck, shaft, shoulder, and stem. Exact terminology is necessary when diagnosing or treating patients with pathology of or near the dens. The authors suggest simplified terminology for describing the parts of the dens that can be used in the future in order to be unequivocal and to avoid confusion when classifying and communicating fractures through its parts.

## Introduction and background

**Figure 1:**
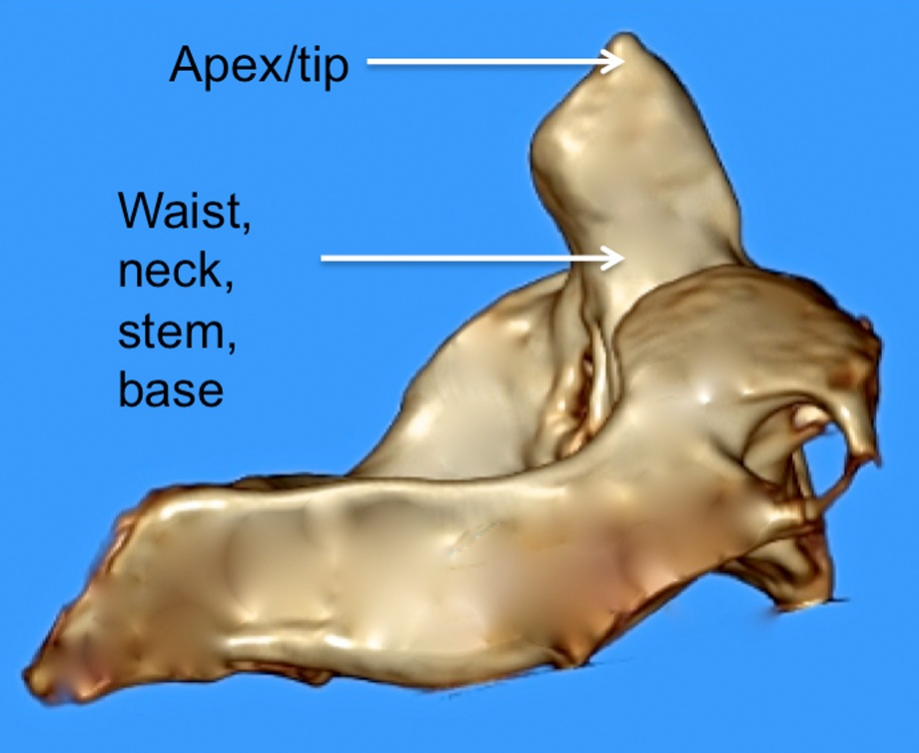
3D reconstructed CT image of C2 noting various terms used to describe parts of the dens.

**Figure 2:**
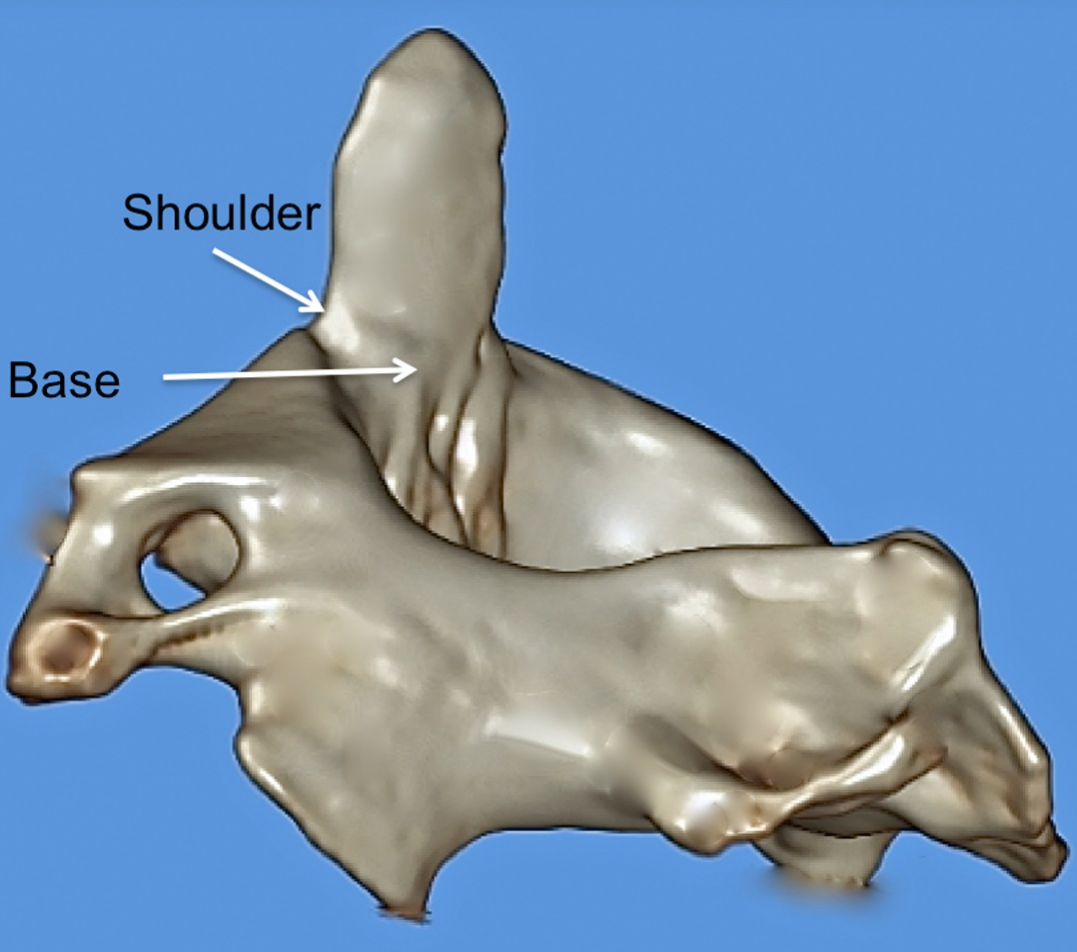
3D reconstructed CT image of C2 noting various terms used to describe parts of the dens.

**Figure 3:**
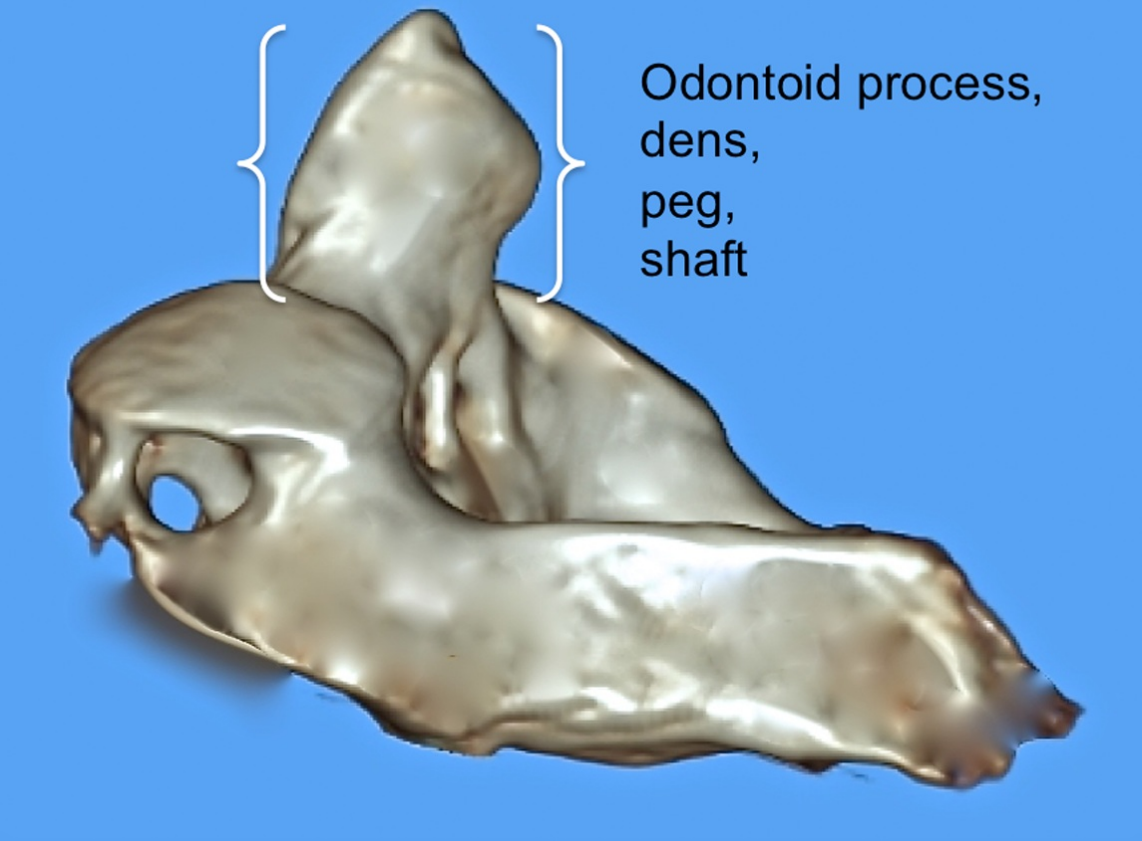
3D reconstructed CT image of C2 noting various terms used to describe parts of the dens.

The dens (odontoid process) of the axis exists as a superior projection from the C2 vertebral body and is conical in shape and serves as an attachment site for the transverse, apical, and alar ligaments [1-2]. A review of the relevant literature reveals a complex nomenclature for the dens, with the same structure being referred to as the odontoid process, the odontoid peg, and the processus epitrophysis [1, 3-4]. Inconsistency also exists in descriptions of the dens itself, with terms such as neck, base, waist, apex, and tip being used to define various parts of the dens (Figures 1-3).

A great deal of structural variability can exist between individuals and their dens and various congenital bony anomalies (e.g., hypoplastic, bifid dens) can alter its structure during the developmental process [5]. Such anomalies may in part contribute to the lack of uniformity observed in the nomenclature of this structure of the upper cervical spine.

## Review

### Anatomical and clinical nomenclature

The official Terminologia Anatomica term for the odontoid process is the dens. This structure’s officially named parts only include an apex and anterior articular and posterior articular facets. However, additionally described parts include a posterior broad groove, which serves as an attachment site for the transverse ligament. The posterolateral surfaces, located just above this groove, serve as an attachment site for the alar ligaments. At its apical end, the dens is pointed in shape and this tip (apex) gives rise to the apical ligament, which extends cephalad to attach onto the basion. The anterior surface of the dens articulates with the anterior arch of the atlas via an ovoid articular facet that is characterized by many vascular foramina [2].

**Figure 4:**
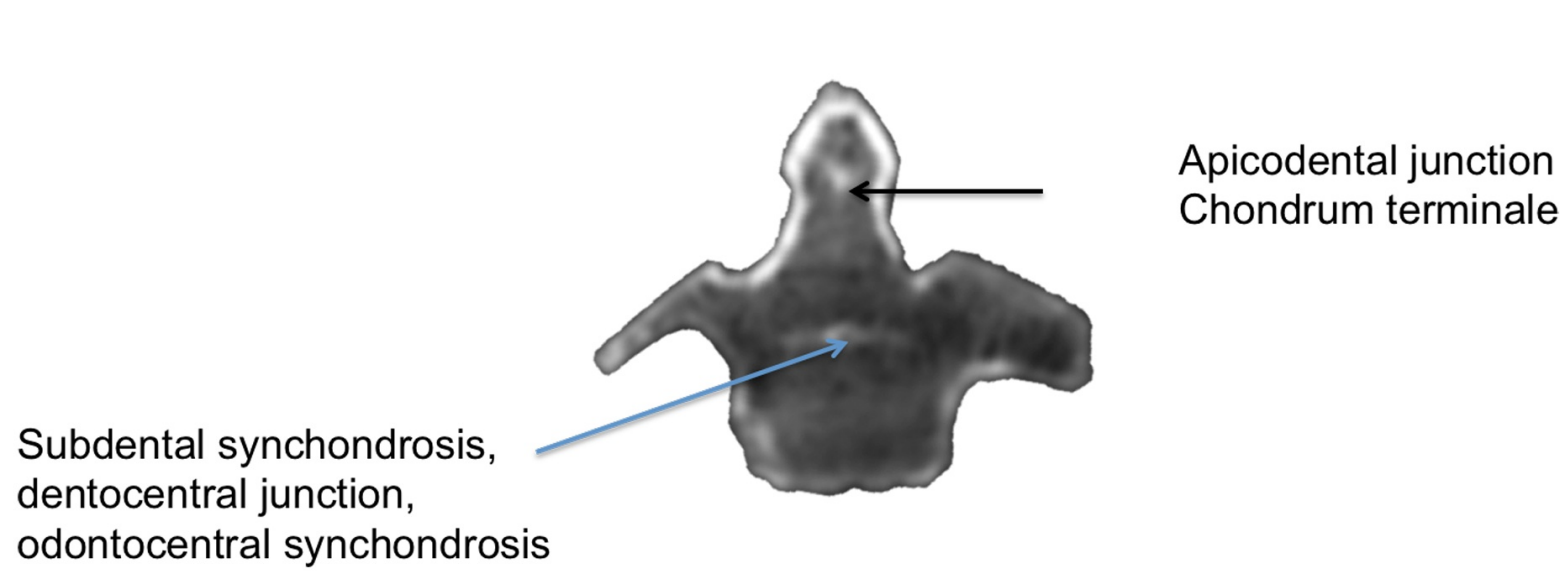
CT scan through a C2 specimen noting the various terms used to describe the fusion points of the dens.

Aydin and Cokluk defined the tip of the dens as extending from the apex of the dens to the apicodental junction and the neck of the dens as the bone between this later junction and a horizontal line connecting the left and right superior articular processes [6]. Caney and Ogden referred to this junction as the chondrum terminale (Figure 4) [7].

When reviewing the literature on the clinical processes that manifest at the dens, a great deal of inconsistency is encountered with respect to the nomenclature used to define its various parts. For example, fractures of the dens are often defined by a classification system proposed by Anderson and D’Alonzo [8]. This classification scheme categorizes fractures of the dens based on the anatomical location of the fracture site. Type I fractures of the dens, the least common in incidence, are defined as occurring at the tip or apex of the dens [5]. Type II fractures, which are the most commonly reported, occur at the base or waist of the dens at the point where it attaches to the C2 vertebral body [8-9]. The waist of the dens has been described differently. For example, Kulkarni, et al. defined the waist of the dens as the part of the dens with the narrowest transverse diameter [10]. However, Vaccaro stated that this part of the dens was the part just superior to the body of the axis [11]. To further confound the true definition of the base of the dens, Jeffreys stated that an os odontoideum is represented by a fracture through the base of the dens [12]. To further confuse these terms, Sherk and Parke used the terms waist and neck interchangeably for the part of the dens where the transverse ligament passes posteriorly [13]. Finally, Aydin and Cokluk described the base as the bone between the subdental synchondrosis and the part of the dens found along a horizontal line connecting the left and right superior articular processes. They termed this synchondrosis the dentocentral junction (Figure 4) [6].

**Figure 5:**
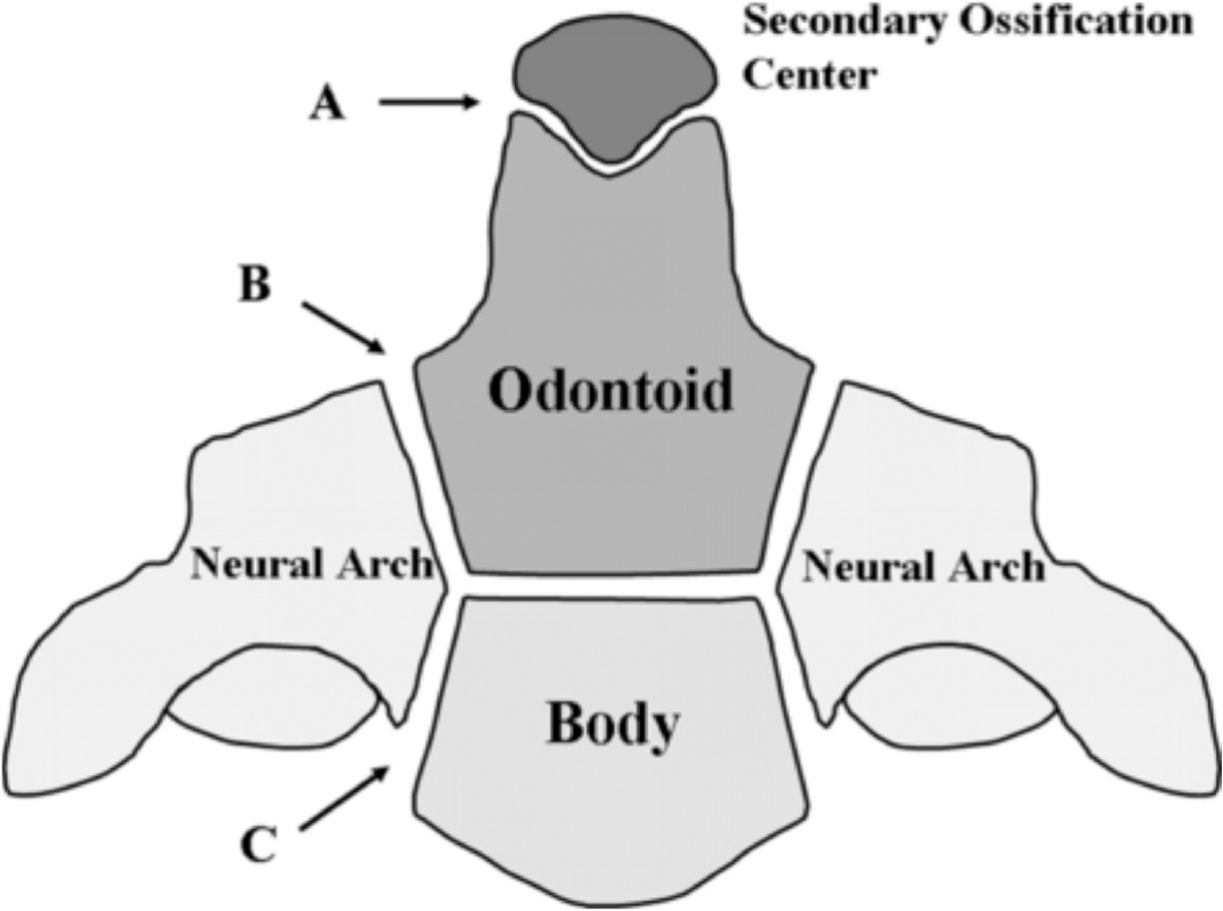
Schematic of ossification centers of the C2 vertebra. The most superior aspect of the dens is shown at A, the junction between the dens and neural arches is shown at B, and the junction between the neural arches and the body of C2 is shown at C. This figure has been modified after Akobo, et al., Childs Nerv Syst (2015) 31:2025–34.

Developmentally, the tip or apex of the dens is derived from a distinct secondary ossification center (cuneiform cartilage), whereas the remainder of the dens arises from laterally located primary ossification centers (Figure 5) [1, 14].

The connection between the tip of the dens and the remainder of the C2 vertebral body may not be fused completely until the beginning of puberty and, until that point, that particular junction may exist as a cartilaginous physis [1]. The connection between the tip of the dens and the base that attaches to the axis has been described as the shaft, the stem, or the neck of the dens in various sources [15-16]. The complex development process of the dens, which is not completed until skeletal maturity is attained during puberty, may contribute in part to the incongruity surrounding the nomenclature of the dens. In addition to the terms tip and apex, other terms used for this region of the dens, although not properly defined, include “upper part of the odontoid process” [17] and “proximal tip of the odontoid process” [18].

**Figure 6:**
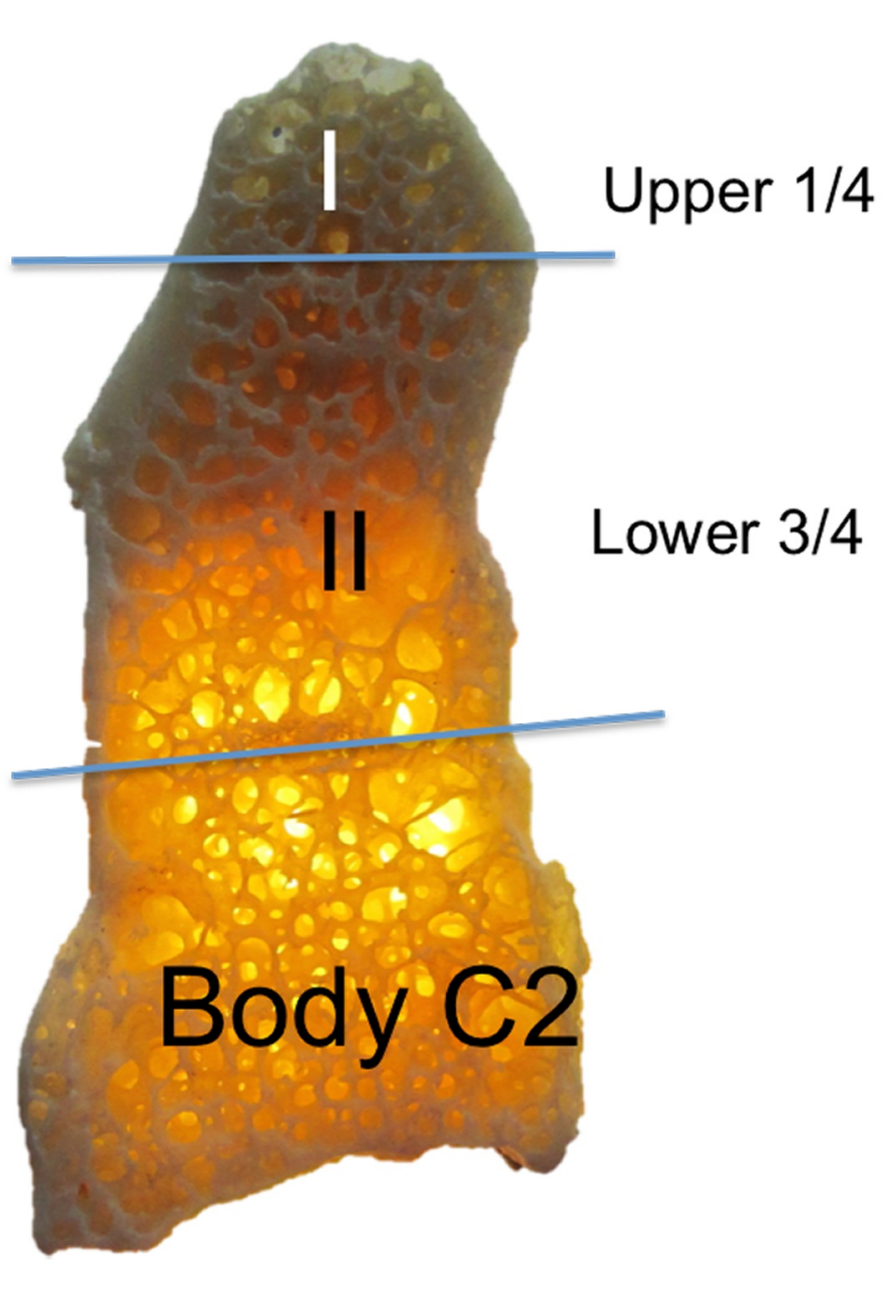
Suggested terminology for simplifying the terms used to describe parts of the dens. Zones I and II are used to describe the upper 1/4th and lower 3/4th of the dens.

**Figure 7:**
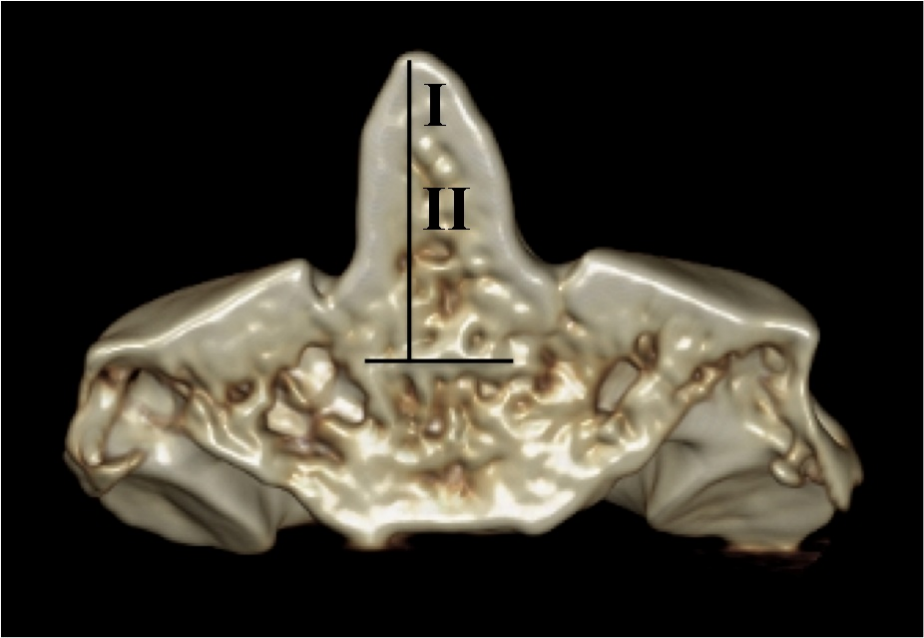
3D CT reconstruction of C2. Zones I and II are based on defining the dens (vertical line) as the midline portion of C2 that fuses with its body at the synchondrosis (horizontal line).

We suggest the following simplified terminology be used for the dens: Zones I and II with Zone I being the upper one-fourth and Zone II being the lower three-fourths of the dens with the dens being defined from its most superior part to its most inferior part down to the site of fusion of the body of C2, i.e., at the synchondrosis. These zones are defined and delineated in the following figures (Figures 6-7).

## Conclusions

In conclusion, a great deal of uncertainty exists with respect to which nomenclature should be used to describe the various parts of the dens. The dens itself is often referred to as the odontoid process or odontoid peg. Its most apical end has been referred to as the tip or apex of the dens, and the apical secondary ossification center that eventually connects with the axis is termed the ossiculum terminale. The part connecting the dens to the axis is described in various sources as the stem, shaft, or neck. Jenkins refers to the shaft of the dens as the part that attaches inferiorly to the centrum of the C2 vertebra at the odontocentral synchondrosis [15]. Finally, the protrusion from which the shaft of the dens sprouts off from the axis at the level of the superior articular facets has been described as the waist or base of the dens. This incongruent and difficult to decipher nomenclature further compounds the uncertainty surrounding the development of the dens and the etiology of the various clinical presentations and pathologies associated with it.

We have proposed a novel naming scheme for the dens that labels Zone I as the upper one-fourth and Zone II being the lower three-fourths of the dens. Consequentially, Zone I is the site of fusion between the primary and secondary ossification centers of the dens and Zone II is the site of fusion to the synchondrosis between the dens and the body of the C2 vertebra. These terms better reflect the embryological derivation of the dens and simplify communication between clinicians regarding fractures or pathology of this part of the C2 vertebra.
